# Supplementary material for: Connected function of PRAF/RLD and GNOM in membrane trafficking controls intrinsic cell polarity in plants
Source: Nat Commun. 2022 Jan 10;13:7. doi: 10.1038/s41467-021-27748-w (PMC8748900; doi:10.1038/s41467-021-27748-w)
Supplement: Supplementary file 1 — Supplementary Information [file 41467_2021_27748_MOESM1_ESM.pdf]

Supplementary Information for

Connected function of PRAF/RLD and GNOM in membrane trafficking controls intrinsic cell polarity in plants

<u>Authors</u>

Lu Wang[1, 2, #], Dongmeng Li[1, 2, #], Kezhen Yang[3, #], Xiaoyu Guo[1], Chao Bian[1, 2, &], Takeshi Nishimura[5], Jie Le[3,4], Miyo Terao Morita[5], Dominique C. Bergmann[6,7], Juan Dong[1,2, *]

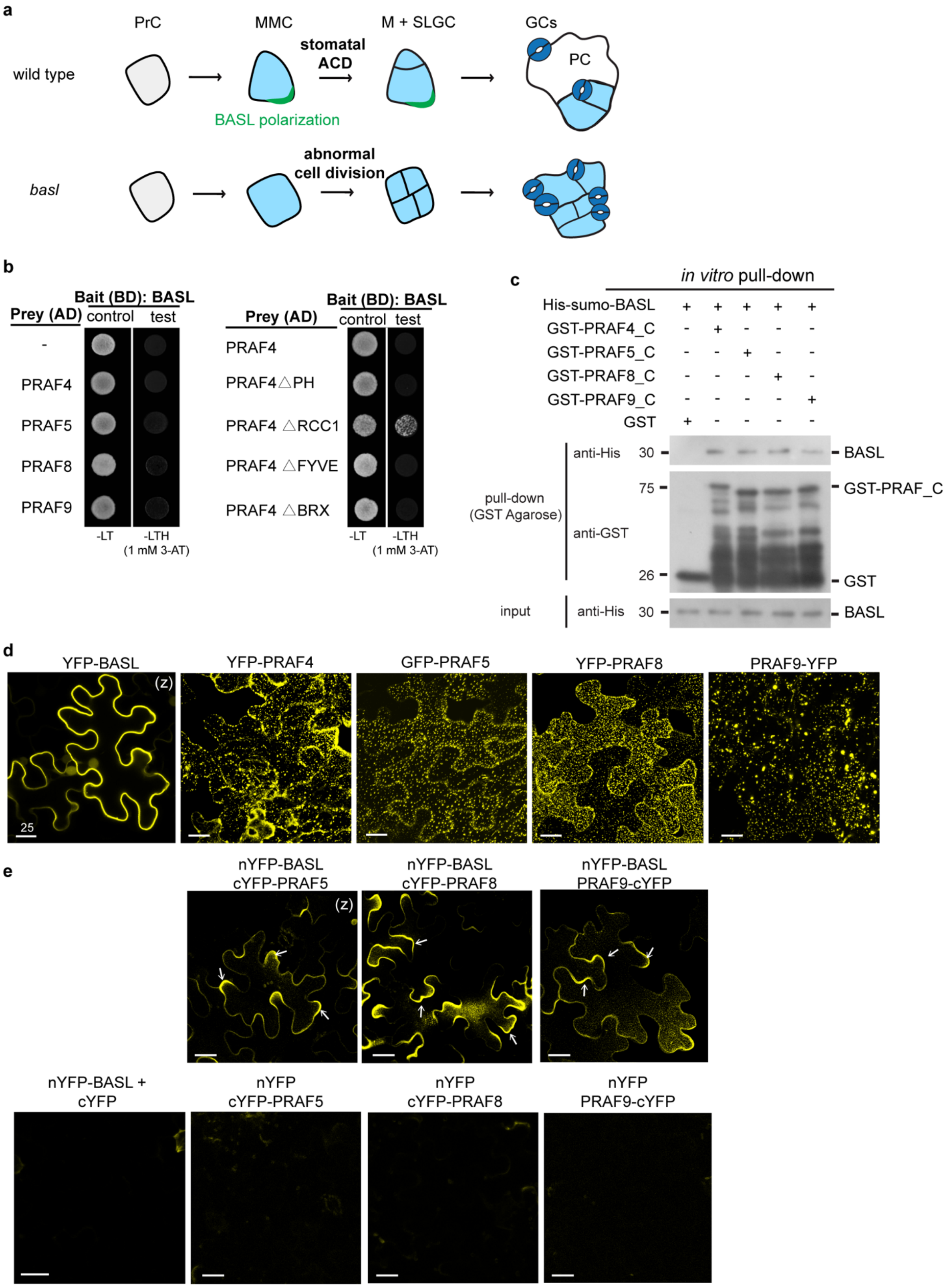
a
PrC
MMC
M + SLGC
GCs
wild type
stomatal
ACD
PC
BASL polarization
basl
abnormal
cell division
b
Prey (AD)
Bait (BD): BASL
control
test
-
PRAF4
PRAF5
PRAF8
PRAF9
-LT
-LTH
(1 mM 3-AT)
PRAF4△PH
PRAF4 △RCC1
PRAF4 △FYVE
PRAF4 △BRX
c
in vitro pull-down
His-sumo-BASL
GST-PRAF4_C
GST-PRAF5_C
GST-PRAF8_C
GST-PRAF9_C
GST
pull-down
(GST Agarose)
anti-His
anti-GST
input
30
75
26
BASL
GST-PRAF_C
GST
d
YFP-BASL
YFP-PRAF4
GFP-PRAF5
YFP-PRAF8
PRAF9-YFP
(z)
25
e
nYFP-BASL
cYFP-PRAF5
nYFP-BASL
cYFP-PRAF8
nYFP-BASL
PRAF9-cYFP
nYFP-BASL +
cYFP
nYFP
cYFP-PRAF5
nYFP
cYFP-PRAF8
nYFP
PRAF9-cYFP

Supplementary Fig. 1 Physical interaction tests for PRAF/RLD with BASL.

a. Graphics show BASL polarization (green) in stomatal asymmetric cell division and development in wild-type *Arabidopsis* (top) and abnormal stomatal division and differentiation in *basl* mutants (bottom).

b. Pairwise yeast two-hybrid assays. Bait, Gal4 DNA-binding domain (BD) fused with BASL. Prey, Gal4 activation domain (AD) fused with full-length PRAF proteins (left) and the domain deletion variants of PRAF4 (right). “-” indicates AD only. “Test” indicates interaction assays using synthetic dropout media (-LTH). “Control” indicates yeast growth using rich media (-LT). 3-AT was supplied to suppress bait auto-activation.

c. *In vitro* pull-down assays using recombinant proteins to test PRAF_C interaction with BASL. GST-PRAF4_C, GST-PRAF5_C, GST-PRAF8_C, and GST-PRAF9_C were used as bait and the amount of His-sumo-BASL being pulled down reflects the interaction between PRAF4/5/8/9_C and BASL. GST alone was used as negative control. The numbers indicate the protein sizes (kDa). Results represent three biological replicates.

d. Confocal images show subcellular localization of indicated proteins (yellow) in *N. benthamiana* leaf epidermal cells. BASL, nuclear and cytoplasmic/PM; PRAF4, PRAF5, PRAF8, and PRAF99, cell membrane and intracellular puncta. (z), z-stacked confocal images, applied throughout the figures. Three independent experiments were performed. Scale bar, 25 µm.

e. BiFC assays in *N. benthamiana* leaf epidermal cells show PRAF5, PRAF8, and PRAF9 interact with BASL, whereas no YFP signals were detected in the negative controls (nYFP co-expressed with cYFP-PRAF5/8/9 and cYFP-co-expressed with nYFP-BASL). Data represent results of three independent experiments. (z), z-stacked confocal image. Scale bar, 25 µm.

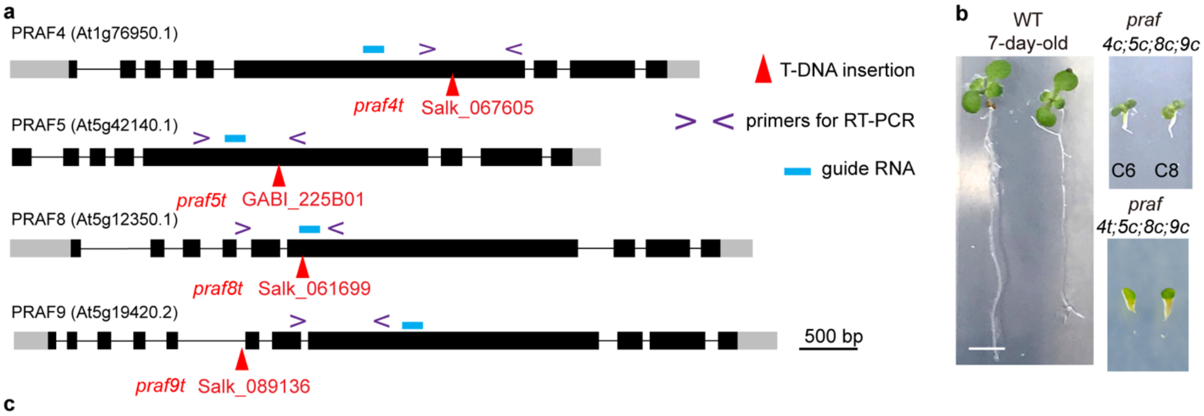

c

praf4c;5c;8c;9c	*#C6*	*#C8*
PRAF4	1-bp insertion	1-bp insertion
PRAF5	1-bp insertion	1-bp insertion
PRAF8	Mix of 3 mutant alleles + WT	Mix of 2 mutant alleles + WT
PRAF9	Mix of 2 mutant alleles + WT	Mix of 2 mutant alleles + WT

praf5c;8c;9c (-Cas9) #18-1			
PRAF4	WT	*#18-1*	tcggacatggtgacaaggatcctcggcttaaacc
		WT	tcggacatggtgacaaggatcctcggcttaaacc
PRAF5	1-bp insertion	*#18-1*	atccggaggacgtcttggccatggaaatgggaaaa
		WT	atccggaggacgtcttggccatgg-aatgggaaaa
PRAF8	2-bp deletion	*#18-1*	gatagcatgcatgga--tatgaggggtatgggcat
		WT	gatagcatgcatggacatatgaggggtatgggcat
PRAF9	2-bp deletion	*#18-1*	acctttggtgttttgggccatg--gacaggaaaag
		WT	acctttggtgttttgggccatggagacaggaaaag

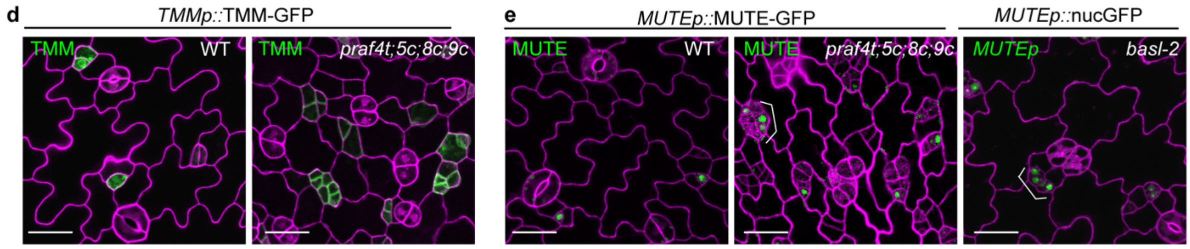

Supplementary Fig. 2 Establishment of CRISPRCRISPR-Cas9 induced *praf* mutant.

a. Gene structures of the PRAF genes. Exon, black box; intron, black line; UTR: grey box.

b. 7-day-old seedlings of wild type (WT), *praf4c;5c;8c;9c*, and *praf4t;5c;8c;9c,* respectively. Scale bar, 0.5 cm.

c. Mutations identified in two somatically chimeric *praf4c;5c;8c;9c* mutants (upper panel, # C6 and # C8) and a Cas9-free *praf5c;8c;9c* mutant (lower panel, # 18-1). Guide RNA sequences are highlighted in blue, and the PAM sequences are in red.

d. Expression pattern of *TMMp*::TMM-GFP (green) in WT and *praf4t;5c;8c;9c*, respectively. Cell outlines are marked by Propidium Iodide (PI) staining (magenta). Scale bars, 25 μm.

e. Expression pattern of *MUTEp*::MUTE-GFP (green) in WT and *praf4t;5c;8c;9c*, respectively, and *MUTEp*::GFP in *basl-2*. Cell outlines are marked by Propidium Iodide (PI) staining (magenta). Scale bars, 25 μm. Data represent results of three independent experiments for (**d**) and (**e**)

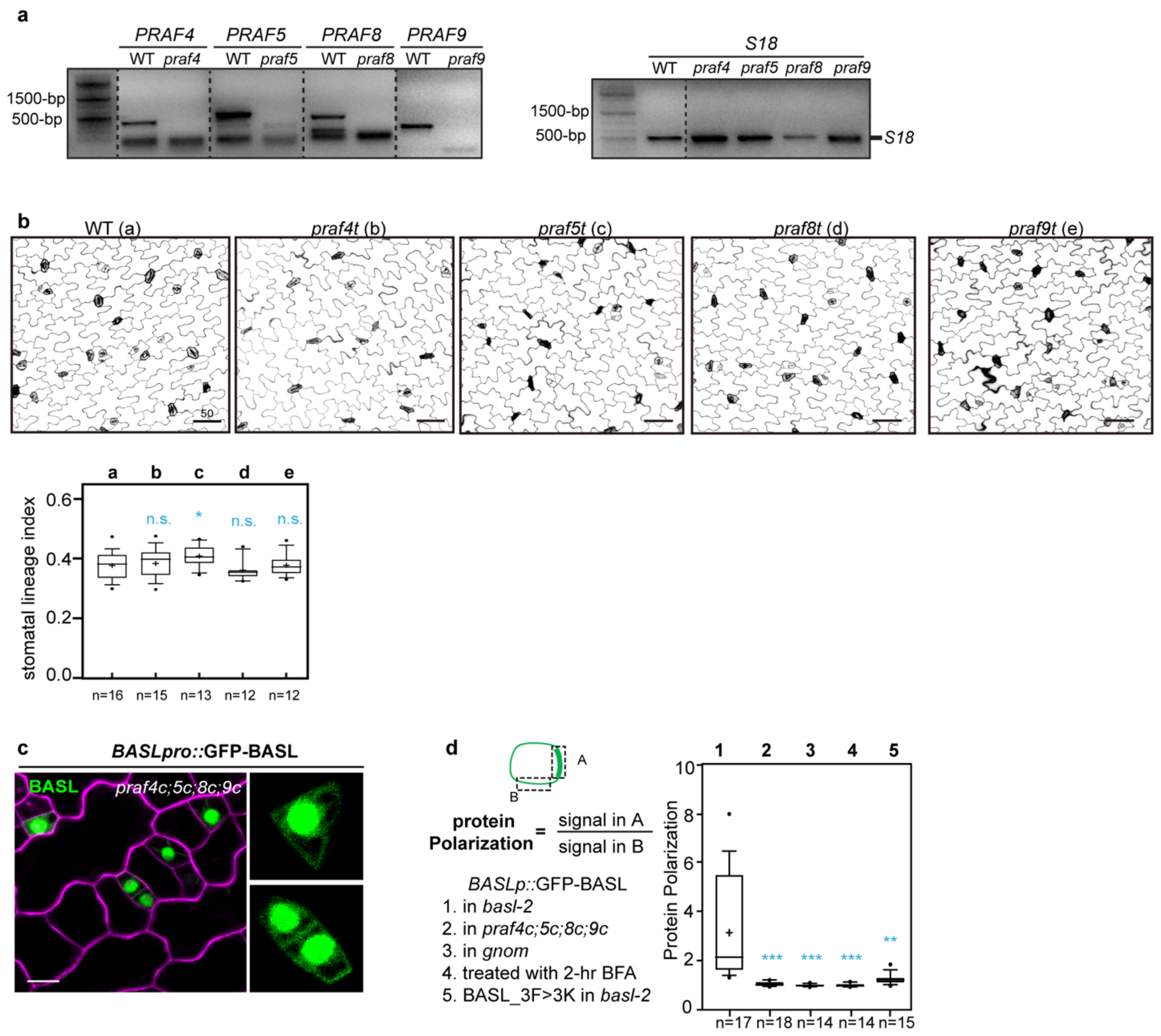

Supplementary Fig. 3 PRAFs are required for stomatal polarity.

a. RT-PCR results evaluate the transcript levels of *PRAF4*, *PRAF5*, *PRAF8*, and *PRAF9* in 5-day-old wild-type seedlings and the respective T-DNA insertional mutant. *S18* was used as an internal reference. Three biological replicates were performed.

b. Upper panels, confocal images show stomatal phenotype of 5-day-old adaxial side cotyledon epidermis of wild type and single mutants of *praf4t*, *praf5t*, *praf8t*, and *praf9t*, respectively. Cell outlines were visualized by PI staining and converted to black/white. Scale bars = 50 μm. Lower panel, quantification of stomata lineage index (ratio of # stomatal lineage cells/ # epidermal cells). n, # cotyledons counted. Student's unpaired *t* tests were used to compare with

wild type. Two-sided P values are 0.6822 (for WT *vs*. *praf4t*), 0.0454 (for WT *vs*. *praf5t*), 0.2924 (for WT *vs*. *praf8t*), and 0.9547 (for WT *vs*. *praf9t*). Three independent experiments were performed.

c. GFP-BASL (green) loses polarity in a *praf4c;5c;8c;9c* mutant. Magenta, cell outlines stained by PI. Three independent experiments were performed. Scale bar, 10 μm.

d. Quantification of GFP-BASL polarization in *basl*-2, *praf4c;5c;8c;9c*, *gnom*, or *basl-2* treated with 70 μM BFA for 2-hr, as well as GFP-BASL_3F>3K in *basl*-2 (mutated for PRAF-binding). Protein polarization is calculated as the ratio of fluorescence intensity measured in segment A (high) *vs*. that in B (low, at the same length). Box plots in (**g**) and (**i**) show first and third quartile (box), median (line) and mean (cross). n, # stomatal lineage cells. Student's unpaired *t* tests were used to compare with GFP-BASL in *basl*-2. Two-sided P values are 0.0009 (for GFP-BASL in *basl-2 vs*. *praf4c;5c;8c;9c*), 0.0007 (for GFP-BASL in *basl-2 vs*. *gnom*), 0.0007 (for GFP-BASL in *basl-2 vs*. *basl-2* with 2hrs' BFA treatment), and 0.002 (for GFP-BASL in *basl-2* *vs*. GFP-BASL_3F>3K in *basl-2*).

Box plots in (**b**) and (**d**) show first and third quartile (box), median (line) and mean (cross). n.s. not significant; * P < 0.05; ** P < 0.005; *** P < 0.001.

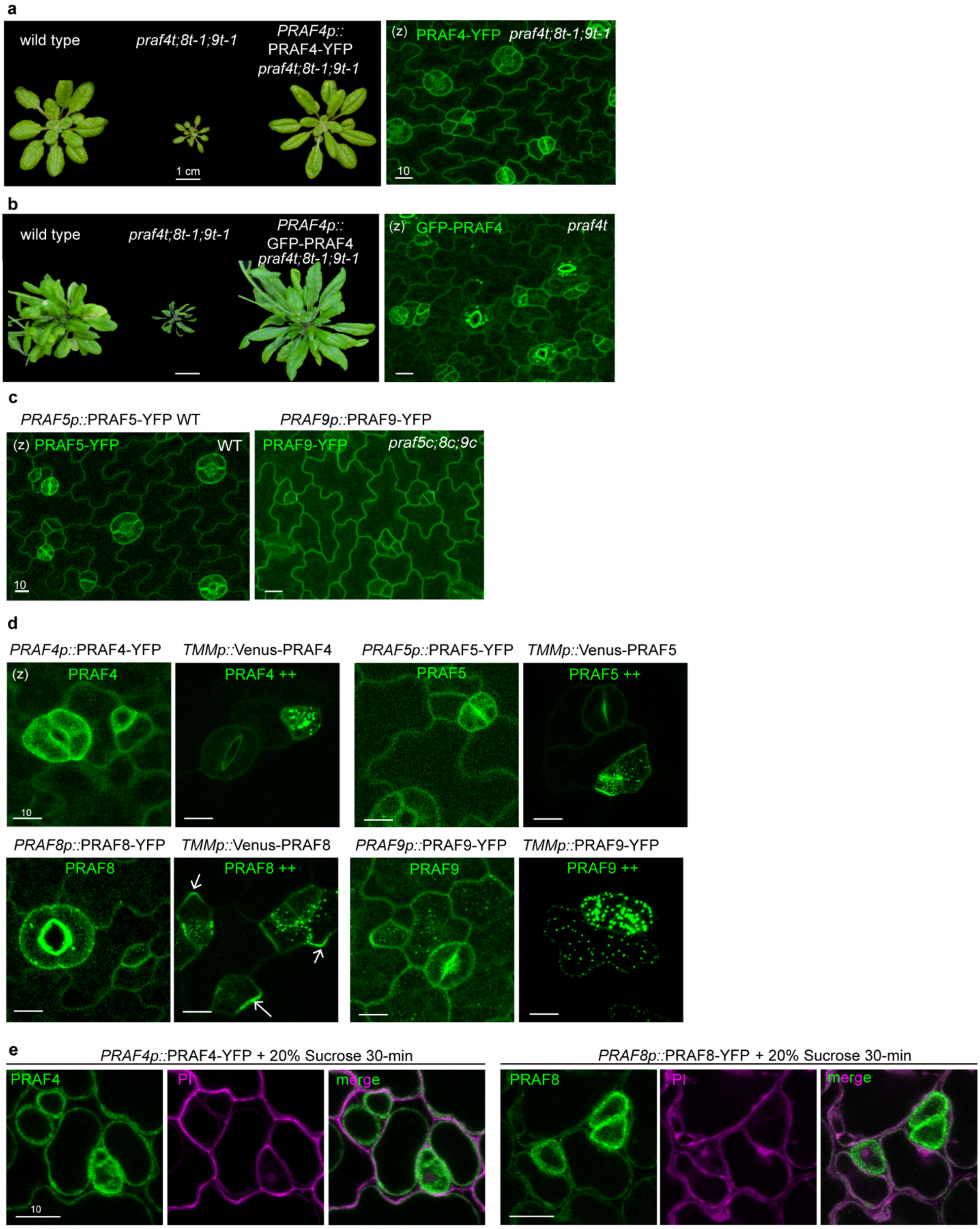

Supplementary Fig. 4 Subcellular localization of the PRAF proteins.

a-b. Growth phenotype of 4-week-old plants (left), including wild-type, *praf4t;8t-1;9t-1*, and *praf4t;8t-1;9t-1* complemented by expressing the native promoter driven PRAF4-YFP (**a**) or GFP-PRAF4 (**b**). Right, protein localization of PRAF4-YFP (**a**) and GFP-PRAF4 (**b**) in 4-day-old cotyledons.

c. Protein localization of PRAF5-YFP (left) and PRAF9-YFP (right) in 4-day-old cotyledons, respectively.

d. Confocal images of 4-day-old adaxial epidermis for localization of YFP/Venus-tagged PRAF4/5/8/9 when expressed at the native level (left) or overexpressed (PRAF4/5/8/9 ++) by the stomatal lineage-specific *TMM* promoter (right). Arrows indicate protein polarization.

e. Plasmolysis experiments demonstrate that PRAF4-YFP (left panel) and PRAF8-YFP (right panel) signals (green) at the cell periphery retract with the plasma membrane that was detached from the cell wall (magenta, stained with PI). Data represent results of three independent experiments.

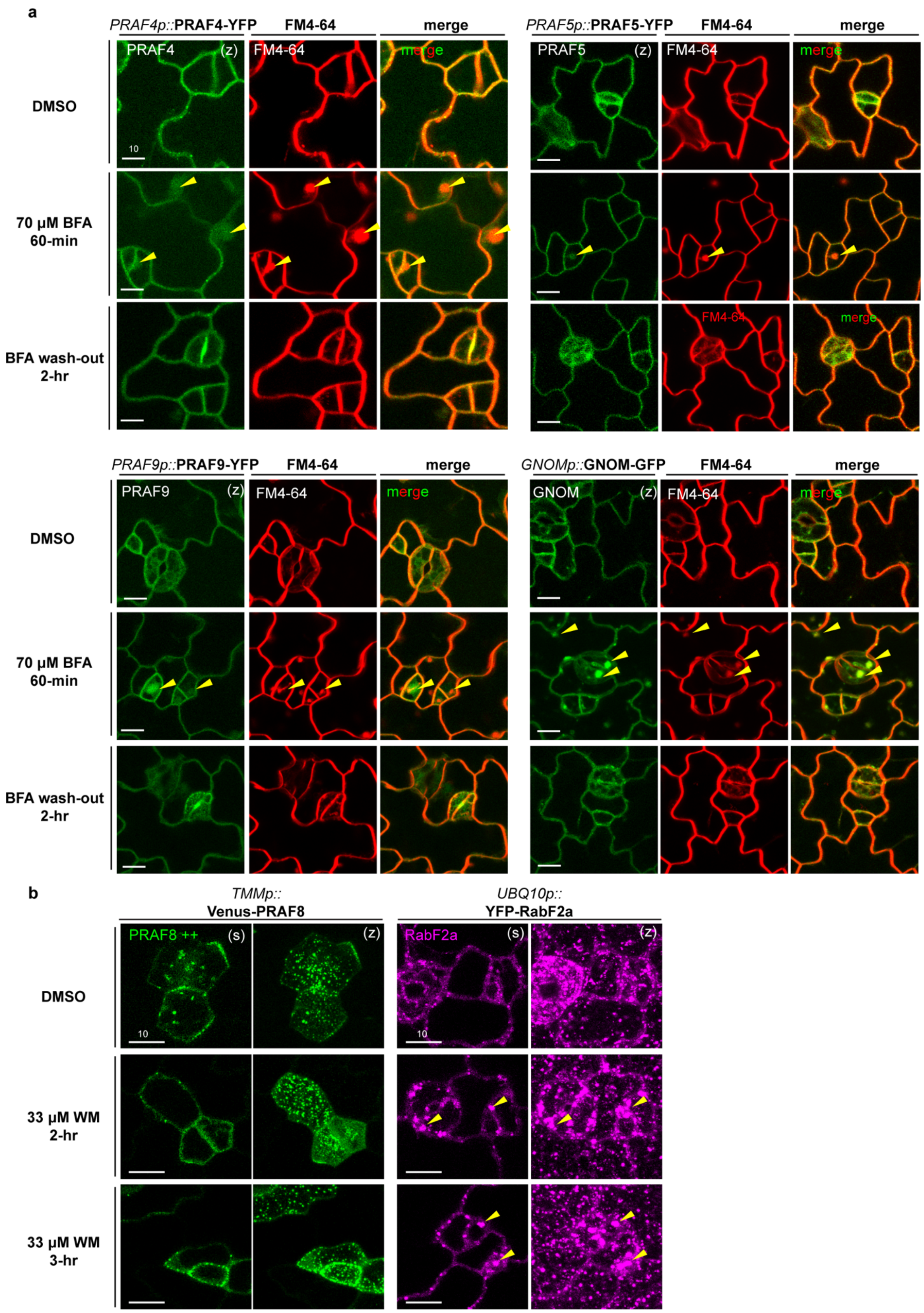
a
PRAF4p::PRAF4-YFP
FM4-64
merge
PRAF5p::PRAF5-YFP
FM4-64
merge
DMSO
70 µM BFA 60-min
BFA wash-out 2-hr
PRAF4
(z)
FM4-64
merge
PRAF5
(z)
10
PRAF9p::PRAF9-YFP
FM4-64
merge
GNOMp::GNOM-GFP
FM4-64
merge
PRAF9
GNOM
b
TMMp:: Venus-PRAF8
UBQ10p:: YFP-RabF2a
PRAF8 ++
(s)
RabF2a
33 µM WM 2-hr
33 µM WM 3-hr

Supplementary Fig. 5 Examination of the subcellular localization of PRAF proteins.

a. PRAF4, PRAF5, PRAF9, and GNOM proteins associate with the endomembrane system. Confocal micrographs show, after the cells stained with the endomembrane tracer FM4-64 for 40-min, PRAF4-YFP, PRAF5-YFP, PRAF9-YFP, and GNOM-GFP (green) overlapped with FM4-64 at the plasma membrane and intracellular vesicles (top panels). After the cells were treated with the Arf GEF inhibitor Brefeldin A (BFA) 70 μM for 60-min (middle panel), internalized PRAF8-YFP overlapped with FM4-64 labelled BFA bodies (yellow arrowheads). Then, 2-hr BFA wash-out with water resulted in greatly reduced intracellular aggregations in these plants.

b. Wortmannin treatment (WM, an inhibitor of PI3K and vacuolar trafficking) of overexpressed PRAF8 (green) in the stomatal lineage cells. The late endosomal/prevacuolar compartment (LE/PVC) marker YFP-RabF2a (magenta) showed enlarged aggregations upon treatment (yellow arrowheads), but no obvious change was observed for Venus-PRAF8.

Data represent results of three independent experiments. (s), single optical section; (z), z-stacked confocal images. Scale bars are as indicated (μm).

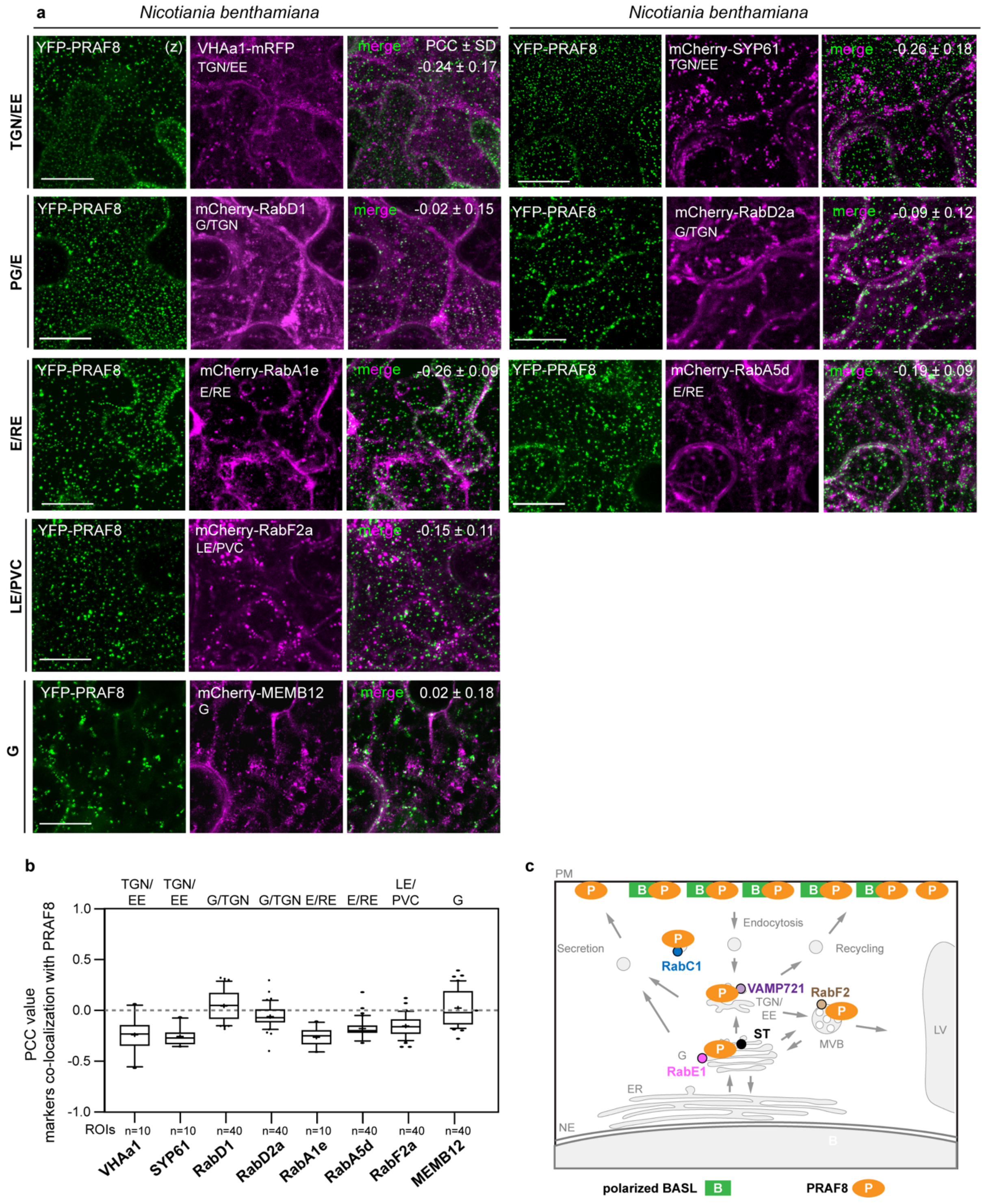

Supplementary Fig. 6 Subcellular localization of PRAF8 in *N. benthamiana* leaf epidermal cells.

a. Representative images of YFP-PRAF8 (green) co-expressed with mCherry/mRFP-tagged endomembrane markers (magenta), including the Golgi (G) marker MEMB12, trans-Golgi network/early endosomal (TGN/EE) markers VHAa1 and SYP61, Golgi/TGN markers RabD1 and RabD2a, endosomal/recycling endosomal (E/RE) markers RabA1e and RabA5d, and late endosomal/prevacuolar compartment (LE/PVC) marker RabF2a. Data represent results of three independent experiments. Protein co-localization rates are calculated as PCC values ± SD (see Methods) and the numbers are shown at the upper-right corner. (z), z-stacked confocal images. Scale bars, 20 μm.

b. Quantification of PCC values for protein co-localization in (**a**). Box plots show first and third quartile (box), median (line) and mean (cross). n, # of ROIs (region-of-interest), in which both proteins are expressed.

c. A hypothetical working model for where PRAF8 and BASL proteins are localized in the stomatal lineage cells.

PM, plasma membrane; NE, nuclear envelope; ER, endomembrane reticulum; G, Golgi; TGN/EE, trans-Golgi network/early endosome; SV, secretory vesicle; RE, recycling endosome; MVB, multivesicular body; LV, lytic vacuole.

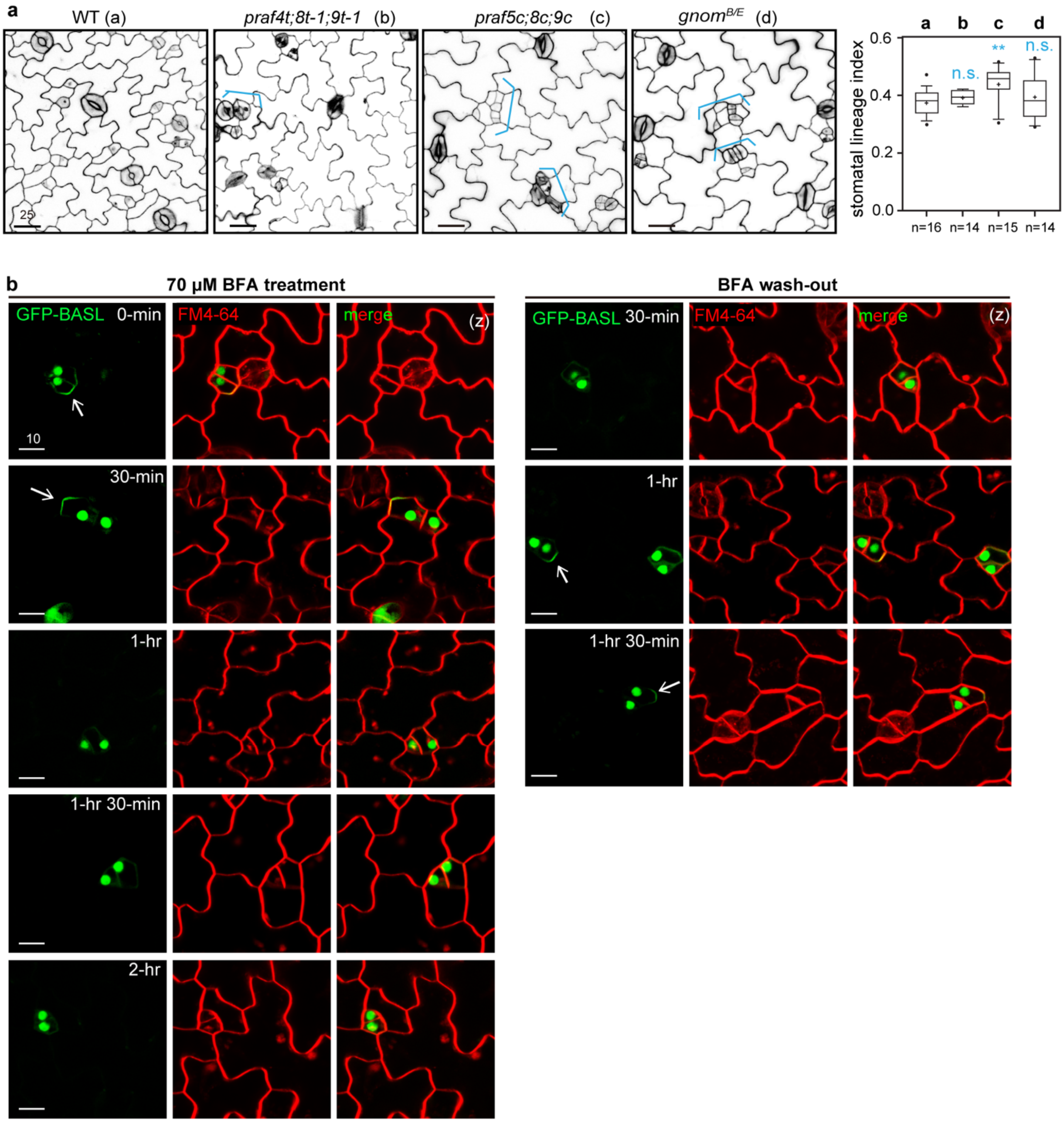

Supplementary Fig. 7 GNOM is required for BASL polarization and stomatal ACD.

a. Left, confocal images show stomatal phenotypes of 5-day-old wild type (WT), *praf4t;8t-1;9t-1*, *praf5c;8c;9c*, and *gnom*$^{B/E}$, respectively. Cell outlines were visualized by PI staining and images were converted to black/white. Blue brackets mark mildly clustered stomatal lineage divisions and guard cells. Scale bars, 25 μm.

Right, quantification of stomata lineage index (ratio of # stomatal lineage cells/ # total epidermal cells). Box plots show first and third quartile (box), median (line) and mean (cross). n, # cotyledons counted. Student's *t* tests were used to compare the mutant with the wild type. Two-sided P values are 0.6822 (for WT *vs*. *praf4t;8t-1;9t-1*), 0.0061 (for WT *vs*. *praf5c;8c;9c*), and 0.4654 (for WT *vs*. *gnom*^{BE}). n.s. not significant; ** P < 0.005.

b. BASL localization changes in responding to BFA treatment (left) and wash-out (right). 3-day-old seedlings expressing GFP-BASL (green) were pre-incubated with 8 μm FM4-64 (red) for 40-min, followed by 70 μm BFA treatment for 0-min, 30-min, 1-hr, 1-hr 30-min, and 2-hr, respectively (left) and BFA wash-out (right) after 30-min, 1-hr, and 1-hr 30min, respectively. Note the disappearance of BASL polarity (white arrows) was observed at 1-hr BFA treatment and the re-establishment of polarity was detected after 1-hr wash-out. Three independent experiments were performed. Scale bars, 10 μm.

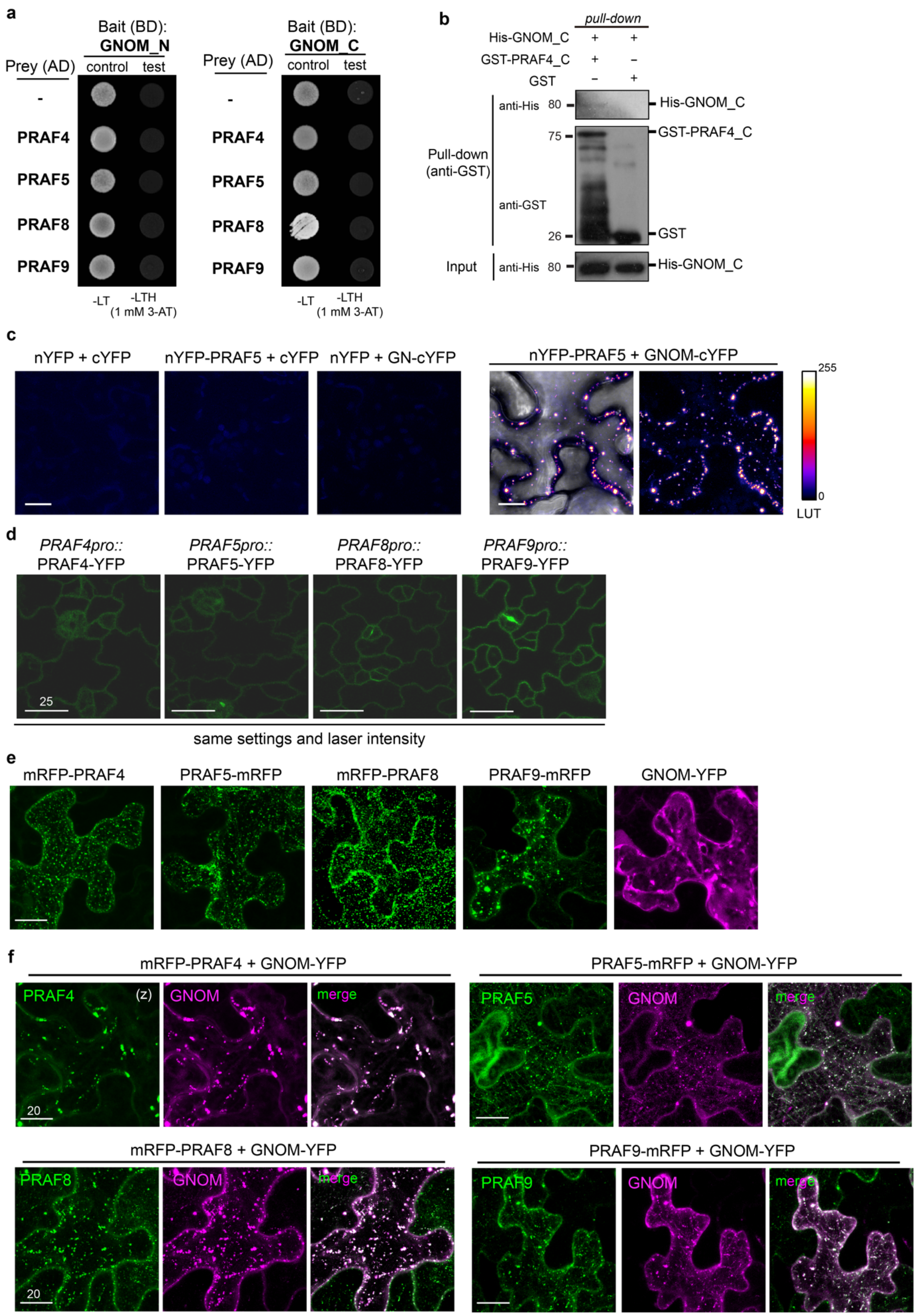

a
Bait (BD):
GNOM_N
Prey (AD)
control
test
-
PRAF4
PRAF5
PRAF8
PRAF9
-LT
-LTH
(1 mM 3-AT)
Bait (BD):
GNOM_C
Prey (AD)
control
test
-
PRAF4
PRAF5
PRAF8
PRAF9
-LT
-LTH
(1 mM 3-AT)
b
pull-down
His-GNOM_C + +
GST-PRAF4_C + –
GST – +
anti-His 80
His-GNOM_C
75
GST-PRAF4_C
Pull-down
(anti-GST)
anti-GST
26
GST
Input
anti-His 80
His-GNOM_C
c
nYFP + cYFP
nYFP-PRAF5 + cYFP
nYFP + GN-cYFP
nYFP-PRAF5 + GNOM-cYFP
255
0
LUT
d
PRAF4pro::
PRAF4-YFP
PRAF5pro::
PRAF5-YFP
PRAF8pro::
PRAF8-YFP
PRAF9pro::
PRAF9-YFP
25
same settings and laser intensity
e
mRFP-PRAF4
PRAF5-mRFP
mRFP-PRAF8
PRAF9-mRFP
GNOM-YFP
f
mRFP-PRAF4 + GNOM-YFP
PRAF4
(z)
GNOM
merge
20
PRAF5-mRFP + GNOM-YFP
PRAF5
GNOM
merge
mRFP-PRAF8 + GNOM-YFP
PRAF8
GNOM
merge
20
PRAF9-mRFP + GNOM-YFP
PRAF9
GNOM
merge

Supplementary Fig. 8 PRAF and GNOM physically interact.

a. Pairwise yeast two-hybrid assays test physical interactions between GNOM_N (left) or GNOM_C (right) with full-length PRAF proteins. Bait, GNOM_N or GNOM_C fused with Gal4 DNA-binding domain (BD). Prey, Gal4 activation domain (AD) fused with PRAF proteins. "-" indicates AD only. "Test", interaction testes performed on synthetic dropout media (-LTH with 3-AT supplemented to suppress bait auto-activity); "Control", yeast growth on rich media (-LT).

b. *In vitro* pull-down assays using recombinant proteins to test the interaction of PRAF4_C with GNOM_C. GST-PRAF4_C was used as bait and the amount of His-GNOM_C being pulled down reflects the interaction strength between PRAF4_C and GNOM_C. GST alone was used as negative control. The numbers show the protein sizes (kDa). Results represent three biological replicates.

c. Three images on the left are negative controls. Right, confocal images show typical BiFC signals generated by co-expression of nYFP-PRAF5 with GNOM-cYFP. Complemented YFP signals were converted to the ImageJ's Fire LUT mode. Scale bars = 20 μm.

d. Confocal images show the comparison of protein expression levels of PRAF4-YFP, PRAF5-YFP, PRAF8-YFP, and PRAF9-YFP (all driven by the respective native promoter). Same confocal settings and laser intensity were applied. Scale bars, 25 μm.

e-f. Confocal images show subcellular localization of individual proteins (**e**), mRFP-tagged PRAF4/5/8/9 (green) and GNOM-YFP (magenta), and co-expression of the PRAF and GNOM proteins (**f**) in *N. benthamiana* leaf epidermal cells. Note the two proteins became highly co-localized when co-expressed (merge). Images are z projected (z). Scale bars, 20 μm. Three independent experiments were performed.

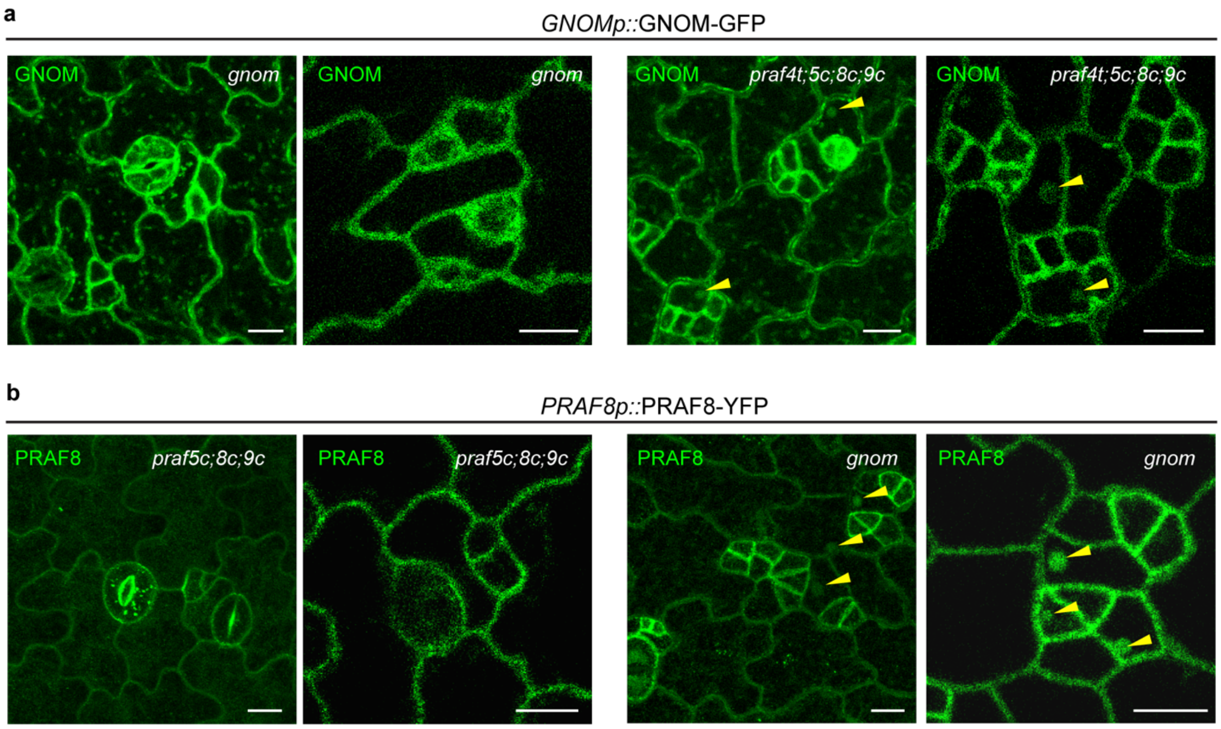

Supplementary Fig. 9 PRAF8 and GNOM are mutually dependent for subcellular localization.

a. Confocal images show differential localization patterns of GNOM-GFP (green) in *gnom*^{T} (two on the left) *vs*. in *praf4t;5c;8c;9c* (two on the right), respectively.

b. Differential localization patterns of PRAF8-YFP (green) in *praf5c;8c;9c* (left panel) *vs*. in *gnom* (right panel). In both (**a**) and (**b**), yellow arrowheads show abnormal aggregation of GNOM and PRAF, respectively. Enlarged views are shown on the right side. Data represent results of three independent experiments. Scale bars, 10 µm.

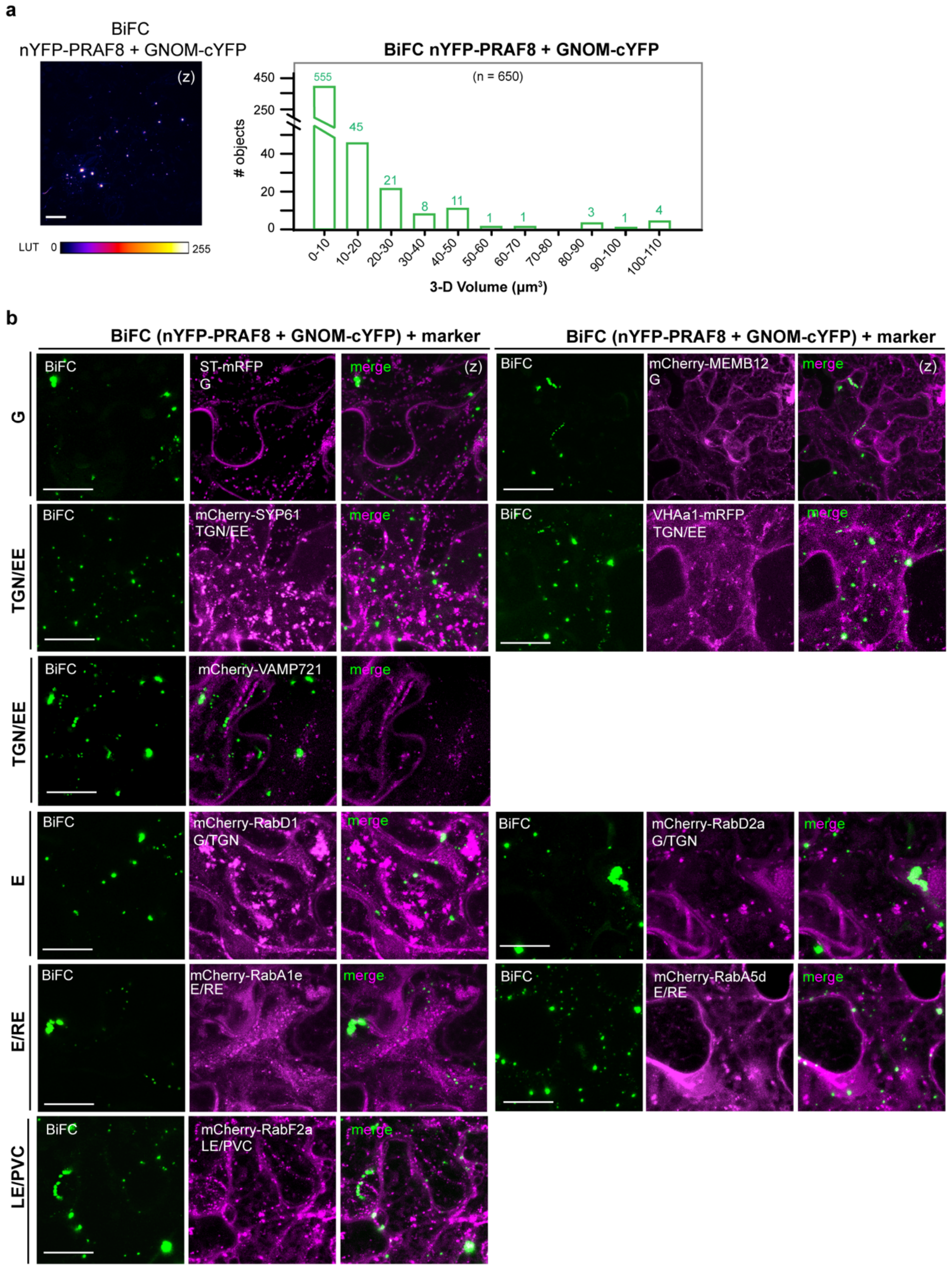

180

181 **Supplementary Fig. 10 BiFC examination of the PRAF8-GNOM interaction in plant cells.**

a. Left, confocal image shows typical BiFC signals generated by co-expression of nYFP-PRAF8 with GNOM-cYFP. Complemented YFP signals were converted to the ImageJ's Fire LUT mode. Scale bars = 50 μm. Right, histograms show quantification of the 3-D volumes of the BiFC compartments. n, # objects in each category.

b. Confocal images show co-expression of mRFP/mCherry-tagged endomembrane markers (magenta) with the BiFC pair (green) of nYFP-PRAF8 and GNOM-cYFP in *N. benthamiana* leaf epidermal cells. The tested markers include the Golgi markers ST and MEMB12, the TGN/EE markers SYP61, VHAa1, and VAMP721, the endosomal markers RabD1 and RabD2a, the E/RE markers RabA1e and RabA5d, and the LE/PVC marker RabF2a. No significant co-localizations were detected in these assays.

Data represent results of three independent experiments. All images are z projected (z). Scale bars, 20 μm.

195

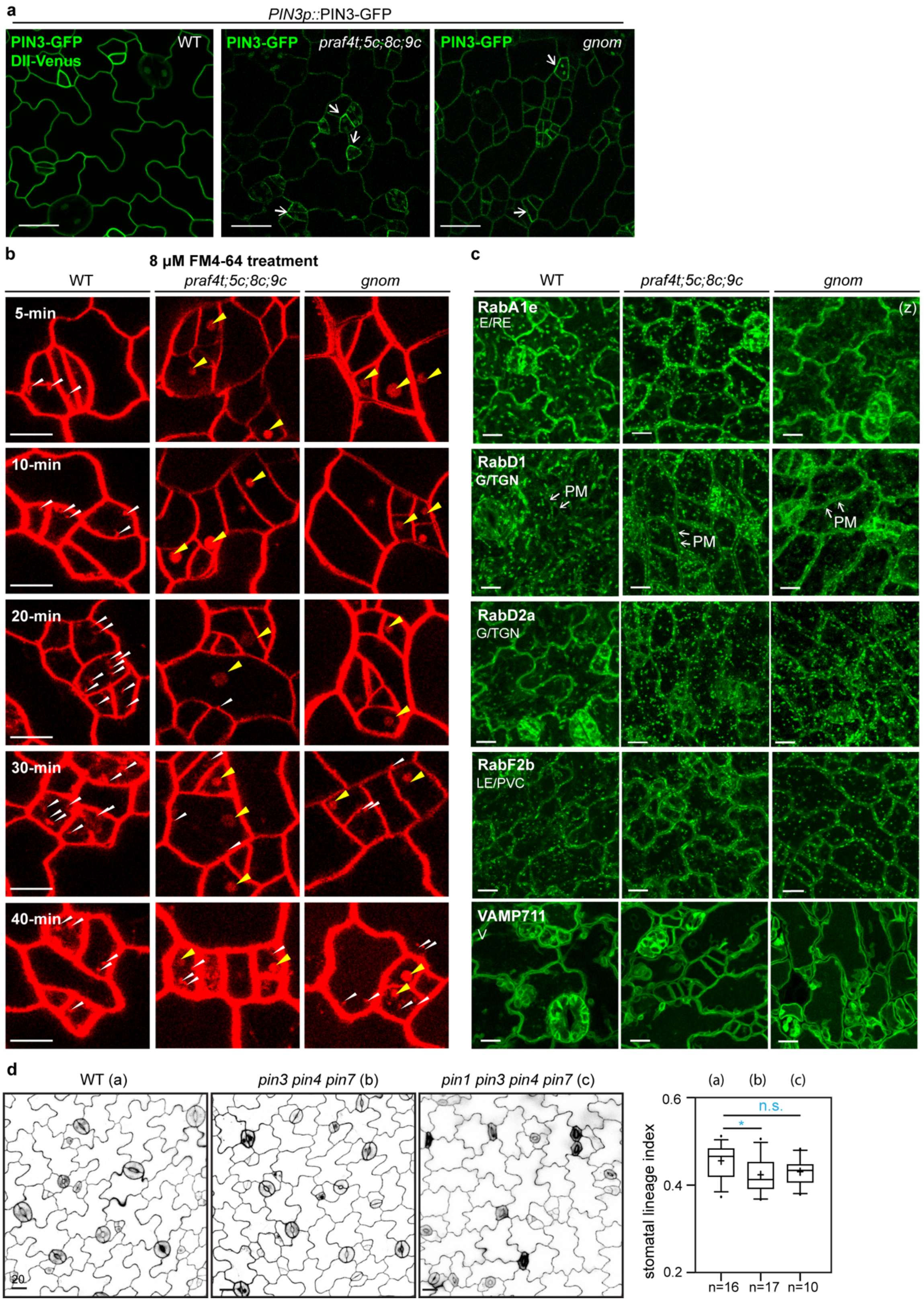

196 **Supplementary Fig. 11 PRAF and GNOM are required for endomembrane trafficking.**

a. The plasma membrane localization of PIN3-GFP became uneven (arrows) in *praf4t;5c;8c;9c* and *gnom* (segregated from *gnom*$^{B/E}$) mutants compared with that in the wild type (WT) in 3-day-old cotyledon epidermal cells. Scale bars, 50 μm.

b. Endocytosis, based on FM4-64 internalization, is lowered in *praf4t;5c;8c;9c* and *gnom* mutants. 3-day-old seedlings were treated with 8 μM FM4-64 (red) for 5-min, 10-min, 20min, 30-min, and 40-min, respectively. White arrowheads indicate typical endocytosed vesicles labeled by FM4-64. Yellow arrowheads mark atypical, large FM4-64 positive aggregations identified in *praf4t;5c;8c;9c* and *gnom* mutants but absent in the WT. Quantification of endocytosis rates and vesicle sizes are shown in Fig. 7c, d, respectively. Scale bars, 10 μm. All images are z-stacked.

c. Differential localization patterns of endomembrane makers (green) in WT (left), *praf4t;5c;8c;9c* (middle) and *gnom* (right). The markers include RabA1e (E/RE), RabD1 (PG/E), RabD2a (PG/E), RabF2b (LE/PVC), and VAMP711 (V). “PM”, the plasma membrane. Note, RabD1 images show strong signals along the plasma membrane in *praf4t;5c;8c;9c* and *gnom,* but hardly observable in the WT. Quantification of endosomal sizes is shown in Fig. 7f. Scale bars, 10 μm.

d. Left panels, confocal images show stomatal phenotypes of 5-day-old wild type (WT), *pin3 pin4 pin7*, and *pin1 pin3 pin4 pin7*, respectively. Cell outlines were visualized by PI staining and images were converted to black/white. Scale bars, 20 μm.

Right panel, quantification of stomata lineage index. Box plots show first and third quartile (box), median (line) and mean (cross). n, # cotyledons counted. Student’s *t* tests were used to compare mutant with the wild type. Two-sided P values are 0.0299 (for WT *vs. pin3 pin4 pin7*), and 0.0785 (for WT *vs. pin1 pin3 pin4 pin7*). n.s. not significant; * $P < 0.05$.

Three independent experiments were performed to generate the figures.

Supplementary Table 1. Primers used in this study

Gene	Primer names	Primer sequences	Purpose
PRAF4	*praf4t*-LP	TGCTAATGCTAAGGCTTCCTG	T-DNA genotyping
	praf4t-RP	CAAAATTGCATGTGGACACAG	
	PRAF4pro-FWD	GGAAATCGAAGGATCAGAAGCTACTAGAAG	*PRAF4* promoter cloning
	PRAF4pro-REV	TCCATGTGAAAAACAGAGCAGAAGAGAAC	
	PRAF4-FWD	ATGGCAGATCTTGTGACCTATAGTAATGCCGAC	Genomic *PRAF4* or *PRAF4* CDS cloning
	PRAF4-REV	GTGAGGAATGTCTTCTTCTTCTTCATCTC	
	PRAF4_sgRNA-FWD	GATTGACATGGTGACAAGGATCCT	*PRAF4* sgRNA cloning
	PRAF4_sgRNA-REV	AAACAGGATCCTTGTCACCATGTC	
	praf4t RT-FWD	CCTGTAGACTGGCTTTTGG	RT-PCR for *PRAF4*
	praf4t RT-REV	TTGGAGTAGCTCTTCGCAT	
	PRAF4_CRISPR-FWD	GGCTTCATGTGGCTTCAGTA	*PRAF4* sgRNA target region genotyping
	PRAF4_CRISPR-REV	CAGATCACCATGCCCTAATCTC	
	PRAF4 △BRX-REV	CTGATTAGTATTGTTAACTCCTGTTTGACTCGAACC	PRAF4 CDS BRX domain deletion cloning
	PRAF4 CDS △PH-FWD	ATGGGCTGGAGTGGC	*PRAF4* CDS PH domain deletion cloning
	PRAF4 CDS △RCC1-REV	TGAAGACATATGGCTGCAGTTTGCATATTTTTGTTGTC	*PRAF4* CDS RCC1 domain deletion cloning
	PRAF4 CDS △RCC1-FWD	ACTGCAGCCATATGTCTTCACAAA	
	PRAF4 CDS △FYVE-REV	TTAGTATCGTTTATCTCAGAAAGACATATGGCTGCAGT	PRAF4 CDS FYVE domain deletion cloning
	PRAF4 CDS △FYVE-FWD	TCTGAGATAAACGATACTAACAGAAGGAACAG	
	PRAF4 CDS BRX-FWD	ATGGTAGAAGCAGAGTGGATCGAACAG	*PRAF4* CDS BRX domain cloning
	PRAF4 CDS PH-FWD	ATGGAACAGGCATTGATTACATTGAAGAAGG	*PRAF4* CDS PH domain cloning
	PRAF4 CDS PH-REV	CCCAGTGGATATTAATGTTTTCAGGC	
	PRAF4 CDS RCC1-FWD	ATGGATGACTCAGATGCTCTAGGT	*PRAF4* CDS RCC1 domain cloning
	PRAF4 CDS RCC1-REV	GTAATTCGATCCGCAAGCAAT	
	PRAF4 CDS FYVE-FWD	ATGCACAAATGGGTGTCTGGTG	*PRAF4* CDS FYVE domain cloning
	PRAF4 CDS FYVE-REV	CACTTTGCTGAGCTTAACATAACA	
	PRAF4 CDS CC-FWD	ATGCTCTATTTCCCGGTGGGTATAG	*PRAF4* CDS coil-coil domain cloning
	PRAF4 CDS CC-REV	GCTGTTTCTTTCGCTGGCTCTA	
	GST-PRAF4 CDS C-FWD	ATGCACAAATGGGTGTCTGGTGCTG	Pull-down assay
PRAF5	PRAF5-FWD	ATGGCAGATCTTGTGAGTTATGGTA	

	PRAF5-REV	GAATTGTGATTGAGATATAGGTGATGTTGCCACAC	Genomic *PRAF5* or *PRAF5* CDS cloning
	praf5t RT-FWD	GGGTGAAGTGCTATGTGAGAA	RT-PCR for *PRAF5*
	praf5t RT-REV	CAATGGTCCTCAAACCAGATAAAG	
	PRAF5_sgRNA-FWD	GATTGGACGTCTTGGCCATGGAAT	*PRAF5* sgRNA cloning
	PRAF5_sgRNA-REV	AAACATTCCATGGCCAAGACGTCC	
	PRAF5_CRISPR-FWD	GGGTGAAGTGCTATGTGAGAA	*PRAF5* sgRNA target region genotyping
	PRAF5_CRISPR-REV	CAATGGTCCTCAAACCAGATAAAG	
	praf5t-LP	TGATGTTCTGATTCCAAAGCC	T-DNA genotyping
	praf5t-RP	ATGAATGGCAGTGGACAAGTC	
	PRAF5 CDS BRX-FWD	ATGGTGGAGGCAGAGTGGATTGAACAATATG	*PRAF5* CDS BRX domain cloning
	PRAF5 CDS BRX-REV	GAATTGTGATTGAGATATAGGTGATGTTGCCACAC	
	GST-PRAF5 CDS C-FWD	ATGCATAAATGGGTTTCTGGTACGGAGC	Pull-down assays
PRAF8	PRAF8pro-FWD	ATTTTTCATAGAGATCGAGACTTTTTTTTTTTTGAAG	*PRAF8* promoter cloning
	PRAF8pro-REV	ACACAAAAAAAAATCTCAACGAAGCTTCTC	
	PRAF8-FWD	ATGTCGAGAAACGGAAGGATGGCT	Genomic *PRAF8* or *PRAF8* CDS cloning
	PRAF8-REV	GCGAGGCAAGTCCTCACTGG	
	praf8t-2 RT-FWD	GAGGTGTGGTTTACTGGTCTTA	RT-PCR for *PRAF8*
	praf8t-2 RT-REV	CACCTTCTCCTATGCCTTCTC	
	PRAF8_sgRNA-FWD	GATTGCATGGACATATGAGGGGTA	*PRAF8* sgRNA cloning
	PRAF8_sgRNA-REV	AAACTACCCCTCATATGTCCATGC	
	PRAF8_CRISPR-FWD	GAGGTGTGGTTTACTGGTCTTA	*PRAF8* sgRNA target region genotyping
	PRAF8_CRISPR-REV	CACCTTCTCCTATGCCTTCTC	
	praf8t-LP	TATGGGCAGTCCTGTCTATGG	T-DNA genotyping
	praf8t-RP	CCAAGCCTTCTCCAATCCTAC	
	praf8t-2-LP	GCATGCATGGACATATGAGG	T-DNA genotyping
	praf8t-2-RP	ATTTGATCCCTTTCCCCAAG	
	myr-PRAF8-FWD	ATGGGCAACAAATGTTGCAGCAAGCGACAGGATACCATGGCCATGTCGAGAAACGGAAGGATGGCT	Myr tagged genomic *PRAF8* cloning
	PRAF8 CDS BRX-FWD	ATGAACGAGAAGGAATGGGTTGAACAAG	*PRAF8* CDS BRX domain cloning
	PRAF8 CDS BRX-REV	GCGAGGCAAGTCCTCACT	
	GST-PRAF8 CDS C-FWD	ATGCACAGGTGGGCATCAGGGAT	Pull-down assays
PRAF9	PRAF9pro-FWD	CACATCACTTTGATCGTCGATCTTGG	*PRAF9* promoter cloning
	PRAF9pro-REV	CACATCACTTTGATCGTCGATCTTGG	
	PRAF9-FWD	ATGTGGAGTGGATTTCGTGATTGCACGAG	

	PRAF9-REV	ATGACCCAAGTCTTCACTTCCTACACC	Genomic *PRAF9* and *PRAF9* CDS cloning
	praf9t-2 RT-FWD	CTGGAGGCTCAGATACGCTA	RT-PCR for *PRAF9*
	praf9t-2 RT-REV	TATACAAATCCCCCGATAGA	
	PRAF9_sgRNA-FWD	GATTGTGTTTTGGGCCATGGAGAC	*PRAF9* sgRNA cloning
	PRAF9_sgRNA-REV	AAACGTCTCCATGGCCCAAAACAC	
	PRAF9_CRISPR-FWD	GATGATGGAGACACATTGGGAG	*PRAF9* sgRNA target region genotyping
	PRAF9_CRISPR-REV	CATAAACAGGACTGCCCATAG	
	praf9t-LP	AGGAACGGACGAATGACGCC	T-DNA genotyping
	praf9t-RP	CAGACACAACCAGAAGAAGATA	
	praf9t-2-LP	CCCATTCCTTTCATGTGACC	T-DNA genotyping
	praf9t-2-RP	TTTCCAGGATCATCTCTGGG	
	PRAF9 CDS BRX-FWD	ATGAACGAAAATGAATGGGTTGAACAAGATGAACCTGG	*PRAF9* CDS BRX domain cloning
	PRAF9 CDS BRX-REV	ATGACCCAAGTCTTCACTTCCTACAC	
	GST-PRAF9 CDS C-FWD	ATGCACAAGTGGGCATCAGGGATG	Pull-down assays
GNOM	GNOM CDS-FWD	ATGGGTCGCCTAAAGTTGCATTCT	*GNOM* CDS cloning
	GNOM CDS-REV	CGAACCAGTTGTGTTTTCAGGAGAAGATAAGC	
	GNOMpro-FWD	TCTAGAGGTGTGTATGATAATG	*GNOM* promoter cloning
	GNOMpro-REV	TTAATCTGCTCAAATCTTCAGCCAG	
	gnom-t-LP	GTGATTTGGGAAATCTGCAAC	T-DNA genotyping
	gnom-t-RP	CTCACCTTTCATTCCTGCTTG	
BASL	BASL_3F-3K-1	CACATCGAAACCACCATCTTCGCGATCCTTGCACTTGACAATCTTTCCAGATTCTTCTGAGACAGGGGGCTG	Site mutation
	BASL_3F-3K-2	CAGCCCCCTGTCTCAGAAGAATCTGGAAAGATTGTCAAGTGCAAGGATCGCGAAGATGGTGGTTTCGATGTG	
Cas9	Cas9-FWD	CCCAAGAGGAACAGCGATAAG	*Cas9* genotyping
	Cas9-REV	TTACTTTTTCTTTTTTGCCTGGC	
CRISPR cloning	pAtU6-F-KpnI	GTGGTACCCATTCGGAGTTTTTGTATCTTGTTTC	PCR amplification of second pAtU6-sgRNA cassette
	sgRNA-R-EcoRI	ACGAATTCGCCATTTGTCTGCAGAATTGGC	
Golgi marker	ST-FWD	ATGATTCATACCAACTTGAAGAAAAAGTTCAGCCTCTT	ST CDS cloning
	ST-REV	GGCCACTTTCTCCTGGCTCTTG	
TGN/EE marker	VAMP721-FWD	ATGGCGCAACAATCGTTGATCTACAGTTTCG	*VAMP721* CDS cloning
	VAMP721-REV	ACACTTAAACCCATGGCAAACTGAGAGCAC	

	VHA-A1-FWD	ATGGAGGAATTCTTAGATAAGTTGCCGCAGATG	*VHA-A1* genomic sequence cloning
	VHA-A1-REV	GATTAAAGCGAAAGAGAAAGGCTTGAACTTGTAACC	
	SYP61-FWD	ATGTCTTCAGCTCAAGATCCATTCTACATTGTTAAAG	*SYP61* genomic sequence cloning
	SYP61-REV	GGTCAAGAAGACAAGAACGAATAGGATGATGAACAA	
