# Supplementary material for: Connected function of PRAF/RLD and GNOM in membrane trafficking controls intrinsic cell polarity in plants
Source: Nat Commun. 2022 Jan 10;13:7. doi: 10.1038/s41467-021-27748-w (PMC8748900; doi:10.1038/s41467-021-27748-w)
Supplement: Supplementary file 3 — Description of Additional Supplementary Files [file 41467_2021_27748_MOESM3_ESM.pdf]

Description of Additional Supplementary Files

Supplementary Data 1. Co-IP and MS identification of PRAF proteins as BASL-interacting proteins in Arabidopsis.

Description:

a. Summary of IP MS data. Results from one of three biological replications.

b. Full list of PRAF peptides identified.
